# 1-Benzyl-1*H*-benzotriazole

**DOI:** 10.1107/S1600536812010951

**Published:** 2012-03-21

**Authors:** P. Selvarathy Grace, Samuel Robinson Jebas, B. Ravindran Durai Nayagam, Dieter Schollmeyer

**Affiliations:** a Department of Chemistry, Popes College, Sawyerpuram 628 251, Tamilnadu, India; b Department of Physics, Sethupathy Govt. Arts College, Ramanathapuram 623 502, Tamilnadu, India; c Institut für Organische Chemie, Universität Mainz, Duesbergweg 10-14, 55099 Mainz, Germany

## Abstract

In the title compound, C_13_H_11_N_3_, the benzotriazole ring system is essentially planar, with a maximum deviation of 0.0173 (18) Å, and forms a dihedral angle of 75.08 (8)Å with the phenyl ring. In the crystal, pairs of weak C—H⋯N hydrogen bonds form inversion dimers. In addition, there are weak C—H⋯π(arene) inter­actions and weak π–π stacking inter­actions, with a centroid–centroid distance of 3.673 (11) Å.

## Related literature

For the biological activity of benzotriazole derivatives, see: Katarzyna *et al.* (2005 ▶); Sarala *et al.* (2007 ▶). For their applications, see: Kopec *et al.* (2008 ▶); Krawczyk & Gdaniec (2005 ▶); Smith *et al.* (2001 ▶); Sha *et al.* (1996 ▶). For a related structure, see: Ravindran *et al.* (2009 ▶). For standard bond-length data, see: Allen *et al.* (1987 ▶).
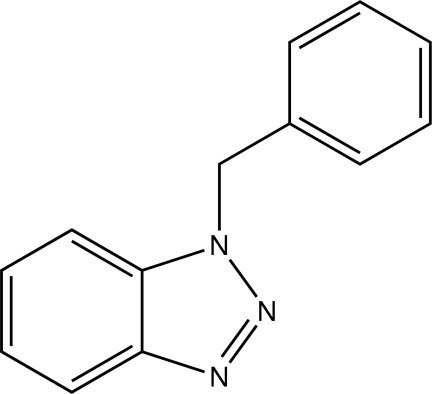


## Experimental

### 

#### Crystal data

C_13_H_11_N_3_
*M* *_r_* = 209.25
Monoclinic, 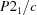

*a* = 11.5734 (10) Å
*b* = 5.9705 (4) Å
*c* = 16.1202 (14) Å
β = 106.490 (4)°
*V* = 1068.07 (15) Å^3^
*Z* = 4
Cu *K*α radiation
μ = 0.64 mm^−1^
*T* = 193 K
0.30 × 0.20 × 0.10 mm

#### Data collection

Enraf–Nonius CAD-4 diffractometer
Absorption correction: ψ scan (*CORINC*; Dräger & Gattow, 1971 ▶; Wiehl & Schollmeyer, 1994 ▶) *T*_min_ = 0.832, *T*_max_ = 0.939
2125 measured reflections
2020 independent reflections
1788 reflections with *I* > 2σ(*I*)
*R*_int_ = 0.108
3 standard reflections every 60 min intensity decay: 2%

#### Refinement

*R*[*F* ^2^ > 2σ(*F* ^2^)] = 0.055
*wR*(*F* ^2^) = 0.138
*S* = 1.12
2020 reflections
145 parameters
H-atom parameters constrained
Δρ_max_ = 0.30 e Å^−3^
Δρ_min_ = −0.30 e Å^−3^

### 

Data collection: *CAD-4 Software* (Enraf–Nonius, 1989 ▶); cell refinement: *CAD-4 Software*; data reduction: *CORINC* (Dräger & Gattow, 1971 ▶; Wiehl & Schollmeyer, 1994 ▶); program(s) used to solve structure: *SHELXS97* (Sheldrick, 2008 ▶); program(s) used to refine structure: *SHELXL97* (Sheldrick, 2008 ▶); molecular graphics: *SHELXTL* (Sheldrick, 2008 ▶); software used to prepare material for publication: *PLATON* (Spek, 2009 ▶).

## Supplementary Material

Crystal structure: contains datablock(s) global, I. DOI: 10.1107/S1600536812010951/lh5426sup1.cif

Structure factors: contains datablock(s) I. DOI: 10.1107/S1600536812010951/lh5426Isup2.hkl

Supplementary material file. DOI: 10.1107/S1600536812010951/lh5426Isup3.cml

Additional supplementary materials:  crystallographic information; 3D view; checkCIF report

## Figures and Tables

**Table 1:** Hydrogen-bond geometry (Å, °) *Cg* is the centroid of the C4–C9 ring.

*D*—H⋯*A*	*D*—H	H⋯*A*	*D*⋯*A*	*D*—H⋯*A*
C8—H8⋯N1^i^	0.95	2.62	3.513 (3)	158
C14—H14⋯*Cg*^ii^	0.95	2.69	3.583 (2)	157

Symmetry codes: (i) 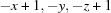; (ii) 
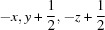
.

**Fig. 1.**
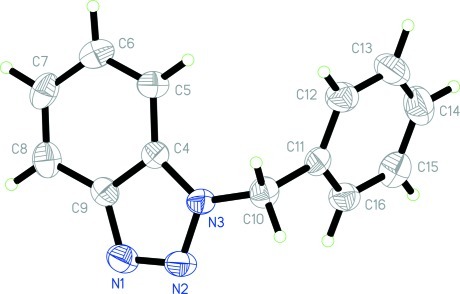
The molecular structure of (I) with displacement ellipsoids are drawn at the 50% probability level.

**Fig. 2.**
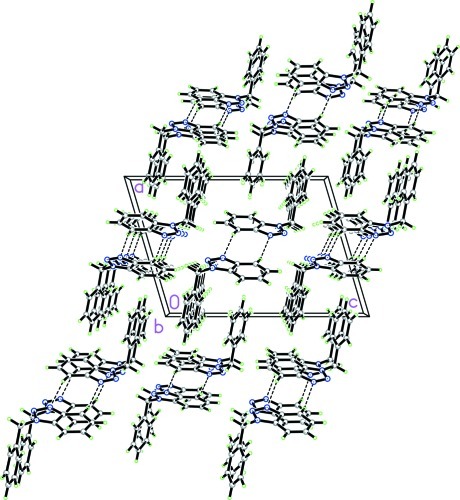
The crystal packing of the title compound, viewed along the b axis. Hydrogen bonds are shown as dashed lines.

## supplementary crystallographic information

### Comment

Benzotriazole derivatives show biological activities such as anti-inflammatory, diuretic, antiviral and are antihypertensive agents (Katarzyna *et al.*, 2005; Sarala *et al.*, 2007). They are used as corrosion inhibitors, antifreeze agents, ultraviolet light stabilizer for plastics and as antifoggants in photography (Krawczyk & Gdaniec, 2005; Smith *et al.*, 2001). *N*-Aryloxy derivatives of benzotriazole have anti-mycobacterial activity (Kopec *et al.*, 2008). Benzotriazole possessing three vicinal N atoms, is used as an antifouling and antiwear reagent (Sha *et al.*, 1996). These applications of benzotriazole compounds prompted us to synthesize the title compound and herein we report the crystal structure.

In (I) (Fig 1), the bond lengths (Allen *et al.*, 1987) and bond angles have normal values. The benzotriazole ring system is essentially planar with a maximum deviation of 0.0173 (18) Å for atom N3. The mean plane of the benzotriazole ring system (N1—N3/C4—C9) forms a dihedral angle of 75.08 (8) Å with the mean plane of the phenyl ring (C11—C16).

In the crystal, pairs of weak C—H···N hydrogen bonds form centrosymmetric dimers (Fig. 2). In addition, there are weak π–π stacking interactions between ring N1-N3/C4/C9 and ring C4—C9(1-*x*, 1-y, 1-*z*) with a centroid-centroid distance of 3.673 (11)Å.

### Experimental

A mixture of the sodium salt of benzotriazole (0.148 g, 1 mmol) benzyl chloride (0.126 g, 1 mmol) in ethanol and water (5 ml) were heated at 333K with continous stirring for 4 h. The mixture was kept aside for slow evaporation. After two weeks crystals of (I) suitable for X-ray diffraction were formed.

### Refinement

H atoms were positioned geometrically [C—H = 0.95 (aromatic) or 0.99 Å (methylene)] and refined using a riding model, with *U*_iso_(H) = 1.2*U*_eq_(C).

### Crystal data

**Table:**

C_13_H_11_N_3_	*F*(000) = 440
*M**_r_* = 209.25	*D*_x_ = 1.301 Mg m^−^^3^
Monoclinic, *P*2_1_/*c*	Cu *K*α radiation, λ = 1.54178 Å
Hall symbol: -P 2ybc	Cell parameters from 25 reflections
*a* = 11.5734 (10) Å	θ = 55–68°
*b* = 5.9705 (4) Å	µ = 0.64 mm^−^^1^
*c* = 16.1202 (14) Å	*T* = 193 K
β = 106.490 (4)°	Block, colourless
*V* = 1068.07 (15) Å^3^	0.30 × 0.20 × 0.10 mm
*Z* = 4	

### Data collection

**Table:**

Enraf–Nonius CAD-4 diffractometer	1788 reflections with *I* > 2σ(*I*)
Radiation source: rotating anode	*R*_int_ = 0.108
Graphite monochromator	θ_max_ = 70.0°, θ_min_ = 4.0°
ω/2θ scans	*h* = 0→14
Absorption correction: ψ scan (*CORINC*; Dräger & Gattow, 1971; Wiehl & Schollmeyer, 1994)	*k* = 0→7
*T*_min_ = 0.832, *T*_max_ = 0.939	*l* = −19→18
2125 measured reflections	3 standard reflections every 60 min
2020 independent reflections	intensity decay: 2%

### Refinement

**Table:**

Refinement on *F*^2^	Primary atom site location: structure-invariant direct methods
Least-squares matrix: full	Secondary atom site location: difference Fourier map
*R*[*F*^2^ > 2σ(*F*^2^)] = 0.055	Hydrogen site location: inferred from neighbouring sites
*wR*(*F*^2^) = 0.138	H-atom parameters constrained
*S* = 1.12	*w* = 1/[σ^2^(*F*_o_^2^) + (0.0628*P*)^2^ + 0.3976*P*] where *P* = (*F*_o_^2^ + 2*F*_c_^2^)/3
2020 reflections	(Δ/σ)_max_ < 0.001
145 parameters	Δρ_max_ = 0.30 e Å^−^^3^
0 restraints	Δρ_min_ = −0.30 e Å^−^^3^

### Special details

**Table:**

Geometry. All esds (except the esd in the dihedral angle between two l.s. planes) are estimated using the full covariance matrix. The cell esds are taken into account individually in the estimation of esds in distances, angles and torsion angles; correlations between esds in cell parameters are only used when they are defined by crystal symmetry. An approximate (isotropic) treatment of cell esds is used for estimating esds involving l.s. planes.
Refinement. Refinement of F^2^ against ALL reflections. The weighted R-factor wR and goodness of fit S are based on F^2^, conventional R-factors R are based on F, with F set to zero for negative F^2^. The threshold expression of F^2^ > 2sigma(F^2^) is used only for calculating R-factors(gt) etc. and is not relevant to the choice of reflections for refinement. R-factors based on F^2^ are statistically about twice as large as those based on F, and R- factors based on ALL data will be even larger.

### Fractional atomic coordinates and isotropic or equivalent isotropic displacement parameters (Å^2^)

**Table:**

	*x*	*y*	*z*	*U*_iso_*/*U*_eq_	
N1	0.42315 (15)	0.1763 (3)	0.37392 (11)	0.0331 (4)	
N2	0.40768 (14)	0.2958 (3)	0.30376 (10)	0.0312 (4)	
N3	0.35886 (13)	0.4957 (3)	0.31508 (10)	0.0255 (4)	
C4	0.34149 (14)	0.5049 (3)	0.39488 (11)	0.0232 (4)	
C5	0.29338 (17)	0.6694 (3)	0.43720 (12)	0.0291 (4)	
H5	0.2638	0.8079	0.4105	0.035*	
C6	0.29158 (18)	0.6177 (4)	0.52002 (13)	0.0345 (5)	
H6	0.2588	0.7231	0.5513	0.041*	
C7	0.33691 (17)	0.4130 (4)	0.55981 (12)	0.0332 (5)	
H7	0.3349	0.3858	0.6174	0.040*	
C8	0.38387 (17)	0.2517 (4)	0.51801 (13)	0.0324 (5)	
H8	0.4147	0.1145	0.5453	0.039*	
C9	0.38385 (15)	0.3007 (3)	0.43252 (12)	0.0256 (4)	
C10	0.33293 (16)	0.6650 (3)	0.24689 (12)	0.0300 (4)	
H10A	0.3745	0.6244	0.2031	0.036*	
H10B	0.3648	0.8114	0.2721	0.036*	
C11	0.19948 (16)	0.6862 (3)	0.20336 (11)	0.0254 (4)	
C12	0.13833 (19)	0.8816 (3)	0.21182 (13)	0.0343 (5)	
H12	0.1804	1.0015	0.2461	0.041*	
C13	0.0167 (2)	0.9019 (4)	0.17048 (15)	0.0425 (5)	
H13	−0.0246	1.0354	0.1769	0.051*	
C14	−0.04510 (19)	0.7302 (4)	0.12006 (15)	0.0434 (6)	
H14	−0.1285	0.7459	0.0912	0.052*	
C15	0.01477 (19)	0.5340 (4)	0.11147 (13)	0.0376 (5)	
H15	−0.0276	0.4148	0.0769	0.045*	
C16	0.13627 (17)	0.5127 (3)	0.15333 (12)	0.0310 (4)	
H16	0.1769	0.3778	0.1478	0.037*	

### Atomic displacement parameters (Å^2^)

**Table:**

	*U*^11^	*U*^22^	*U*^33^	*U*^12^	*U*^13^	*U*^23^
N1	0.0330 (9)	0.0294 (9)	0.0363 (9)	0.0057 (7)	0.0088 (7)	−0.0014 (7)
N2	0.0301 (8)	0.0319 (9)	0.0331 (9)	0.0056 (7)	0.0113 (7)	−0.0050 (7)
N3	0.0241 (7)	0.0282 (8)	0.0260 (8)	0.0022 (6)	0.0099 (6)	−0.0012 (6)
C4	0.0192 (8)	0.0264 (9)	0.0247 (9)	−0.0029 (7)	0.0072 (6)	−0.0010 (7)
C5	0.0314 (9)	0.0262 (10)	0.0323 (10)	0.0010 (8)	0.0133 (8)	−0.0016 (8)
C6	0.0370 (10)	0.0374 (11)	0.0331 (10)	−0.0026 (9)	0.0164 (8)	−0.0062 (9)
C7	0.0329 (10)	0.0446 (12)	0.0232 (9)	−0.0092 (9)	0.0097 (7)	0.0026 (8)
C8	0.0303 (9)	0.0327 (11)	0.0322 (10)	−0.0045 (8)	0.0059 (8)	0.0069 (8)
C9	0.0213 (8)	0.0250 (9)	0.0294 (9)	−0.0012 (7)	0.0055 (7)	0.0002 (7)
C10	0.0289 (9)	0.0354 (11)	0.0288 (9)	−0.0016 (8)	0.0130 (8)	0.0051 (8)
C11	0.0285 (9)	0.0303 (10)	0.0212 (8)	0.0005 (7)	0.0129 (7)	0.0064 (7)
C12	0.0410 (11)	0.0291 (10)	0.0356 (11)	0.0025 (9)	0.0155 (9)	0.0016 (9)
C13	0.0417 (12)	0.0384 (12)	0.0501 (13)	0.0146 (10)	0.0174 (10)	0.0082 (10)
C14	0.0303 (10)	0.0581 (15)	0.0403 (12)	0.0073 (10)	0.0074 (9)	0.0125 (11)
C15	0.0372 (11)	0.0433 (12)	0.0319 (10)	−0.0057 (9)	0.0090 (8)	−0.0009 (9)
C16	0.0345 (10)	0.0298 (10)	0.0315 (10)	0.0027 (8)	0.0137 (8)	0.0010 (8)

### Geometric parameters (Å, º)

**Table:**

N1—N2	1.306 (2)	C10—C11	1.509 (2)
N1—C9	1.376 (2)	C10—H10A	0.9900
N2—N3	1.354 (2)	C10—H10B	0.9900
N3—C4	1.358 (2)	C11—C16	1.387 (3)
N3—C10	1.460 (2)	C11—C12	1.391 (3)
C4—C9	1.388 (3)	C12—C13	1.382 (3)
C4—C5	1.399 (3)	C12—H12	0.9500
C5—C6	1.376 (3)	C13—C14	1.376 (3)
C5—H5	0.9500	C13—H13	0.9500
C6—C7	1.411 (3)	C14—C15	1.388 (3)
C6—H6	0.9500	C14—H14	0.9500
C7—C8	1.373 (3)	C15—C16	1.382 (3)
C7—H7	0.9500	C15—H15	0.9500
C8—C9	1.409 (3)	C16—H16	0.9500
C8—H8	0.9500		
			
N2—N1—C9	108.00 (16)	N3—C10—H10A	109.2
N1—N2—N3	108.93 (15)	C11—C10—H10A	109.2
N2—N3—C4	110.05 (15)	N3—C10—H10B	109.2
N2—N3—C10	120.85 (15)	C11—C10—H10B	109.2
C4—N3—C10	129.11 (15)	H10A—C10—H10B	107.9
N3—C4—C9	104.45 (16)	C16—C11—C12	118.97 (18)
N3—C4—C5	132.60 (17)	C16—C11—C10	120.57 (17)
C9—C4—C5	122.94 (17)	C12—C11—C10	120.46 (18)
C6—C5—C4	115.70 (18)	C13—C12—C11	120.2 (2)
C6—C5—H5	122.2	C13—C12—H12	119.9
C4—C5—H5	122.2	C11—C12—H12	119.9
C5—C6—C7	121.96 (19)	C14—C13—C12	120.5 (2)
C5—C6—H6	119.0	C14—C13—H13	119.8
C7—C6—H6	119.0	C12—C13—H13	119.8
C8—C7—C6	122.18 (18)	C13—C14—C15	119.8 (2)
C8—C7—H7	118.9	C13—C14—H14	120.1
C6—C7—H7	118.9	C15—C14—H14	120.1
C7—C8—C9	116.38 (18)	C16—C15—C14	119.8 (2)
C7—C8—H8	121.8	C16—C15—H15	120.1
C9—C8—H8	121.8	C14—C15—H15	120.1
N1—C9—C4	108.56 (16)	C15—C16—C11	120.71 (19)
N1—C9—C8	130.63 (18)	C15—C16—H16	119.6
C4—C9—C8	120.80 (18)	C11—C16—H16	119.6
N3—C10—C11	111.88 (15)		
			
C9—N1—N2—N3	−0.1 (2)	N3—C4—C9—C8	177.98 (16)
N1—N2—N3—C4	−0.4 (2)	C5—C4—C9—C8	−2.1 (3)
N1—N2—N3—C10	179.31 (15)	C7—C8—C9—N1	−179.62 (19)
N2—N3—C4—C9	0.78 (18)	C7—C8—C9—C4	1.9 (3)
C10—N3—C4—C9	−178.94 (17)	N2—N3—C10—C11	106.88 (18)
N2—N3—C4—C5	−179.12 (18)	C4—N3—C10—C11	−73.4 (2)
C10—N3—C4—C5	1.2 (3)	N3—C10—C11—C16	−67.4 (2)
N3—C4—C5—C6	−179.41 (18)	N3—C10—C11—C12	113.53 (19)
C9—C4—C5—C6	0.7 (3)	C16—C11—C12—C13	−0.3 (3)
C4—C5—C6—C7	0.8 (3)	C10—C11—C12—C13	178.74 (17)
C5—C6—C7—C8	−1.0 (3)	C11—C12—C13—C14	−0.5 (3)
C6—C7—C8—C9	−0.4 (3)	C12—C13—C14—C15	0.8 (3)
N2—N1—C9—C4	0.6 (2)	C13—C14—C15—C16	−0.3 (3)
N2—N1—C9—C8	−178.05 (18)	C14—C15—C16—C11	−0.5 (3)
N3—C4—C9—N1	−0.84 (19)	C12—C11—C16—C15	0.9 (3)
C5—C4—C9—N1	179.07 (17)	C10—C11—C16—C15	−178.22 (17)

### Hydrogen-bond geometry (Å, º)

Cg is the centroid of the C4–C9 ring.

**Table:**

*D*—H···*A*	*D*—H	H···*A*	*D*···*A*	*D*—H···*A*
C8—H8···N1^i^	0.95	2.62	3.513 (3)	158
C14—H14···*Cg*^ii^	0.95	2.69	3.583 (2)	157

Symmetry codes: (i) −*x*+1, −*y*, −*z*+1; (ii) −*x*, *y*+1/2, −*z*+1/2.
